# Microbial alterations in the lungs of children with chronic pulmonary aspiration

**DOI:** 10.3389/fped.2025.1520487

**Published:** 2025-04-11

**Authors:** Nadine Freitag, Tobias Wienemann, Thi Minh Thao Lea Nguyen, Thomas Höhn, Julia Kristin, Dirk Schramm

**Affiliations:** ^1^ Department of General Pediatrics, Neonatology and Pediatric Cardiology, Medical Faculty, University Hospital Düsseldorf, Düsseldorf, Germany; ^2^ Institute of Medical Microbiology and Hospital Hygiene, Medical Faculty, University Hospital Düsseldorf, Düsseldorf, Germany; ^3^ Department of Otorhinolaryngology, Medical Faculty, University Hospital Düsseldorf, Düsseldorf, Germany

**Keywords:** chronic pulmonary aspiration, pediatric, microbiome, dysphagia, bronchoalveolar lavage, pseudomonas aeruginosa, immune response, respiratory health

## Abstract

**Introduction:**

Chronic pulmonary aspiration (CPA), a common complication of pediatric dysphagia, poses significant risks to respiratory health, potentially leading to bronchiectasis and permanent lung damage. Despite its clinical impact, the mechanisms underlying aspiration-related lung injury remain unclear. This study investigates the microbial alterations in the lungs of children with CPA and their association with immune responses.

**Methods:**

We conducted a retrospective analysis of bronchoalveolar lavage fluid (BALF) from children diagnosed with CPA and from controls without swallowing difficulties. Data were collected from patients who underwent bronchoscopy at the University Hospital Düsseldorf between 2010 and 2022. Microbial profiles—including bacterial, viral, and fungal components—and immune cell populations, were assessed to explore the relationship between microbial colonization and immune response in CPA.

**Results:**

The study included 371 children, of whom 48 had CPA. The CPA group exhibited altered microbial colonization, with an increased prevalence of *Pseudomonas aeruginosa* and Enterobacterales. While the presence of mixed upper respiratory flora did not differ significantly between groups, pathogenic bacteria were more frequently detected in CPA patients. Notably, total leukocyte counts were elevated in the CPA group, yet neutrophilia was absent.

**Discussion:**

Our findings suggest that children with CPA have a distinct lung microbial composition, characterized by increased colonization of Enterobacterales and *P. aeruginosa*. These microbial changes may contribute to the pathogenesis of aspiration-related lung disease. Further research is needed to determine whether microbial colonization directly contributes to lung damage in and assess long-term consequences.

**Conclusion:**

Microbial dysbiosis in the lungs of children with CPA underscores the need for targeted interventions to prevent or mitigate aspiration-related lung disease. A deeper understanding of microbial colonization in CPA may pave the way for novel therapeutic strategies and improved patient outcomes.

## 1 Introduction

Swallowing disorders, commonly referred to as dysphagia, are increasingly prevalent in the pediatric population, particularly as medical advances have improved the survival of infants born prematurely or with severe health conditions. Chronic pulmonary aspiration (CPA), a frequent complication of dysphagia, occurs when food, fluids, gastric contents, or saliva are repeatedly aspirated into the lungs. CPA poses a significant threat to respiratory health, often leading to conditions such as bronchiectasis, which can result in permanent lung damage and reduced lung function (1).

Studies estimate that up to 10% of pediatric pneumonia hospitalizations are due to aspiration-related pneumonia, with affected children experiencing higher mortality rates (2) and more frequent ICU admissions compared to those with community-acquired pneumonia (3).

Despite the clear clinical impact of CPA, the underlying mechanisms that lead to lung damage remain poorly understood. Bronchoalveolar lavage (BAL) is frequently used in diagnosing lower respiratory tract infections, including CPA, and offers valuable insights into lung microbiology and immune responses (4). Previous studies have suggested that children with CPA exhibit altered lung microbiota, with a shift toward oropharyngeal flora (5) and an increased prevalence of mixed upper respiratory flora (6). However, findings have been inconsistent, and the extent to which microbial colonization contributes to lung injury remains unclear. In addition to microbial dysbiosis, immune responses in CPA have been described as variable, with some studies reporting neutrophilic inflammation indicative of acute immune activation, while others suggest chronic inflammatory patterns dominated by lymphocytic infiltration (7, 8). However, these studies often involved small, heterogeneous cohorts, limiting their generalizability. Moreover, it is not well understood whether the immune responses observed in CPA are directly related to microbial colonization or are a secondary consequence of repeated aspiration.

This study aims to address these gaps by characterizing the microbial and immune profiles in the lungs of children with CPA compared to those without swallowing difficulties. By analyzing a well-defined pediatric cohort, this study seeks to determine whether CPA is associated with distinct microbial signatures and whether these alterations correlate with specific immune responses.

## 2 Methods

### 2.1 Study design and population

In this retrospective study, medical charts of children between 0 and 18 years of age who underwent bronchoscopy and BAL between 2010 and 2022 at the Children's Hospital of the University Hospital Düsseldorf, Germany were reviewed. Inclusion criteria were the completion of both examinations and a complete documentation. Children that suffered from CPA were the group of interest, children with no history or clinical evidence of swallowing difficulties were considered the control group. CPA was defined as the recurrent inhalation of food, liquids, saliva, or gastric contents into the lower respiratory tract due to impaired airway protection during swallowing. It was either diagnosed by a clinical evaluation of swallowing (CSE) performed by our speech and language pathologist and/or by an instrumental procedure, most commonly a fiberoptic endoscopic evaluation of swallowing (FEES). FEES was performed in tandem by our lead pediatric pulmonologist and our speech and language pathologist. Endoscopic signs of gastroesophageal reflux included erythema, edema, erosions, ulcerations, friability, esophageal strictures, nodularity, and the presence of a hiatal hernia. As this was a retrospective study, informed consent was waived. The study was approved by the ethical review board of the Heinrich-Heine University Düsseldorf, Germany (study number 5199/2015-905).

### 2.2 Procedure

Patients were clinically evaluated for acute infections and blood was drawn for a complete blood count and determination of C-reactive protein. Elective bronchoscopy was only performed if there was no acute infection (except for emergency bronchoscopies or bronchoscopies for the detection of pathogens in cases of suspected radiological findings). The bronchoscopy and the BAL—if medically indicated—were performed according to international standards (9). In brief, introduction of the bronchoscope through one nostril using lubricant. Assessment of anatomical conditions in the nasopharynx, evaluation of vocal cord movements with light propofol sedation and anesthesia of the vocal cords with local lidocaine before intubation. Assessment of anatomy, airway stability, and mucosal texture down to the segmental level. Placement of the bronchoscope in a wedge position and triple irrigation of the lung with warmed saline solution (1 ml per kg body weight). BAL fluid (BALF) was sent to our laboratories for microbiological cultures, multiplex PCR-based genotyping of respiratory viruses, fungal cultures, molecular genetics for atypical pneumonia pathogens and FACS analysis of cell components. Bronchoscopies were performed as in-patient procedures; the length of the stay was usually one night.

### 2.3 Statistical analysis

Demographic and clinical data were retrieved from medical records, including gestational and corrected age, gender, pre-existing medical conditions, blood test results, bronchoscopy findings, BAL laboratory results, nutritional status, and swallowing diagnostics. Descriptive statistics (means ± standard deviations for continuous variables, frequencies and percentages for categorical variables) were calculated. A *p*-value ≤0.05 was considered statistically significant. Statistical analysis followed a targeted approach, with hypothesis testing conducted selectively for variables that demonstrated potential differences in descriptive statistics. This focused testing strategy was employed to minimize the risk of Type I errors while maintaining statistical power for key research questions.

Categorical data were compared using the Chi-Square test of independence, and means were compared using Student's *t*-test for independent samples. All analyses were performed using IBM SPSS Statistics, version 29.

## 3 Results

### 3.1 Sample profile

A total of 371 children who underwent bronchoscopy with BAL were included in the study, of whom 48 were diagnosed with CPA. The remaining 323 served as the control group. Detailed characteristics of the CPA group are presented in Table 1.

**Table 1:** Characterization of the CPA group.

Nutritional status	Number	Percentage
Oral feeding	29	61
Partial oral and tube feeding^a^	3	6
Tube feeding^b^	13	27
Parenteral feeding	1	2
Unknown	2	4
Total	48	100
Respiratory status		
Tracheal canula	2	4
Diagnosis of CPA		
CSE	24	50
Barium swallow	4	8
FEES	20	42
Total	48	100.0

CSE, clinical evaluation of swallowing; FEES fiberoptic endoscopic evaluation of swallowing.

Diagnosis of CPA was either made by CSE or instrumentally in addition to CSE.

^a^ Nasogastric tube (2×), Percutaneous Endoscopic Eastrostomy (PEG) tube (1×).

^b^ Nasograstric tube (9×), PEG tube (4×).

The overall sample had a mean age of 6.4 ± 4.78 years, with 204 children (55%) being male. Demographic data and pre-existing condition specialties for both the CPA and control groups are shown in Table 2.

**Table 2:** Comparison between CPA and control group.

Variable	CPA group	Control group	Significance (*P*-value^a^)
Total number of patients	48	323	
Age (years)	4.8 ± 4.5	6.7 ± 4.8	.011*
Male	56.3%	54.8%	.878
Pre-existing conditions	100%	91%	.022*
Prematurity (*n*; %)	13; 27.1	31; 9.6	.001*
Gestational age (*n*; mean ± SD)	12; 33.69 ± 2.44	27; 32.08 ± 2,96	.242
Neurology (*n*; %)	29; 59.2	23; 7.1	<.001*
Gastroenterology (*n*; %)	19; 39.6	47; 14.6	<.001*
Syndrome	20	14;	<.001*
Genetically confirmed	12;	11;	<.001*
Not confirmed	8;	3;	<.001*
Cardiology	21;	24;	<.001*
Orthopedics	10;	12;	<.001*
Pulmonology	44;	278;	.285
Protracted bacterial bronchitis	5;	49;	.384
Oncology	3;	23;	.826
Psychology	3;	7;	.103
Rheumatology/immunology	4;	27;	.995
Endocrinology	4;	14;	.229
Nephrology	3;	9;	.206
Urology/gynecology	2;	3;	.074

Differences between both groups in regard to age, gender and the presence of pre-existing conditions.

^a^ *P*-value was computed by Chi-Square test for independence for categorial data and by Student’s *t*-test or Welch’s test for continuous data.

* Significant *P*-values.

The number of performed bronchoscopies and lung lavages varies across the inclusion period, however the relative amount of CPA and control cases are comparable in each year (see Supplementary file 1).

### 3.2 Bronchoscopy findings

Airway anatomical alterations were identified in 127 patients (34.2%). Of these, 7 (1.9%) had an aberrant tracheal bronchus, 2 (0.5%) had laryngeal clefts, 2 had tracheal fistulas, 2 had papillomas, and 1 (0.3%) had a cyst in the right main bronchus. The remaining anomalies were unclassified, mostly located in the trachea (11.3%) or right middle lobe (8.6%).

Altered airway anatomy was significantly more common in children with swallowing disorders than in controls [54.1% vs. 31.3%; *χ*^2^(1) = 9.73, *p* = 0.0018]. Signs of inflammation were present in 58.3% of patients with swallowing disorders and 65.9% of those without, most commonly generalized along the airway (47.9% vs. 47.4%). Tracheomalacia was more prevalent in the swallowing disorder group (6.3% vs. 4.3%). Detailed findings are available in Supplementary Tables 1–3.

### 3.3 Microorganisms

**Figure 1:**
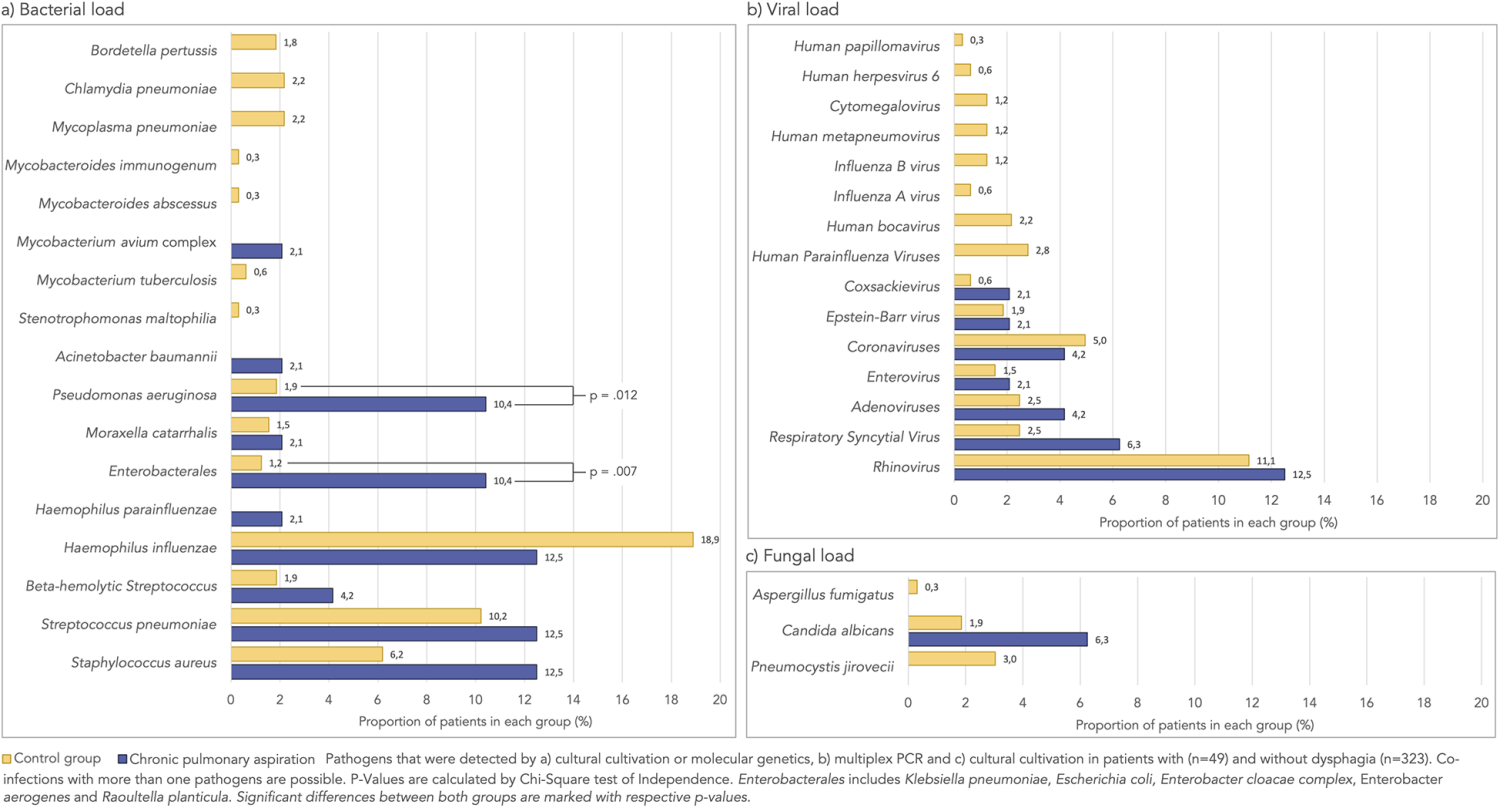
Percentage of patient's in each group whose bronchoalveolar lavage fluid was contaminated by the respective pathogen. **(a)** Bacterial load. **(b)** Viral load.

Bacteria of the physiological oral and pharyngeal microbiota (also often labeled as mixed upper respiratory flora) were detected in 324 (87.3%) patients with no significant differences between both groups (87.6% in the control group vs. 85.5% in the CPA group). Figure 1 displays the bacterial, viral and fungal load that was detected in the BALF of patients with and without CPA. Overall, pathogens were detected more frequently in CPA patients than in controls, though this difference was not statistically significant [68.8% of CPA patients and 54.8% of controls, *χ*^2^(1) = 3.31, *p* = 0.069]. *Pseudomonas aeruginosa* and *Enterobacterales* were significantly more often found in children with CPA.

While bacterial pathogens showed significant differences between groups, viral and fungal components did not differ significantly. Specifically, respiratory viruses were detected in 18.8% of CPA patients compared to 15.5% in controls (*p* = 0.57), and fungal elements were present in 12.5% of CPA patients vs. 9.6% in controls (*p* = 0.53).

### 3.4 Multidrug resistant bacteria

Multi drug resistant (MDR) or pan-resistant (XDR) bacteria were very rarely discovered in our cohort. *Staphylococcus aureus* was discovered in the BALF of six patients with CPA (12.5%) and 20 patients without swallowing disorder (6.2%). None of the strains was resistant to Methicillin (MRSA).

Gram-negative bacteria which are classified as *3MRGN* (*MDR gram-negative bacteria)* or *4MRGN (XDR gram-negative bacteria)* according to the KRINKO (German Commission for Hospital Hygiene and Infection Prevention) classification were detected in ten patients with CPA (20.8%) and ten patients with regular swallowing (3.1%). In the CPA group, one gram-negative bacterium was resistant to all four lead antibiotics (*4MRGN)* and in the control group, one gram-negative bacterium was resistant to three out of four lead antibiotics (*3MRGN)*.

The antibiotic susceptibility testing was based on the breakpoints of the European Committee on Antimicrobial Susceptibility Testing (EUCAST).

### 3.5 Cytological analysis of BALF

**Figure 2:**
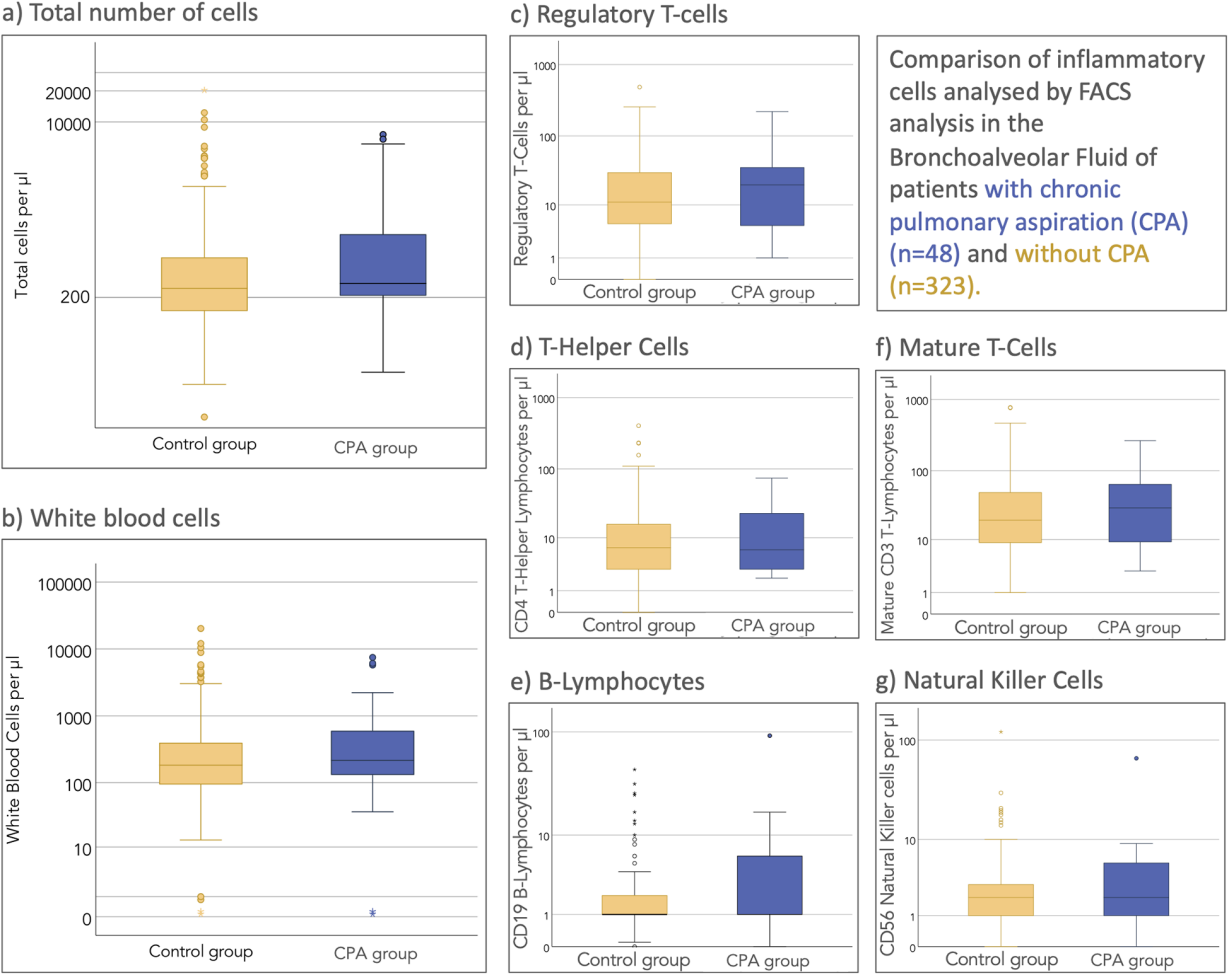
Cytological FACS analysis of the cell populations in the bronchoalveolar lavage fluid. **(a)** Total number of cells. **(b)** White blood cells. **(c)** Regulatory T-cells. **(d)** T-helper cells. **(e)** B-lymphocytes. **(f)** Mature T-cells. **(g)** Nature killer cells.

White blood cells and their subgroups were compared between patients with and without CPA. Figure 2 illustrates the total and white blood cell counts between both groups.

**Figure 3:**
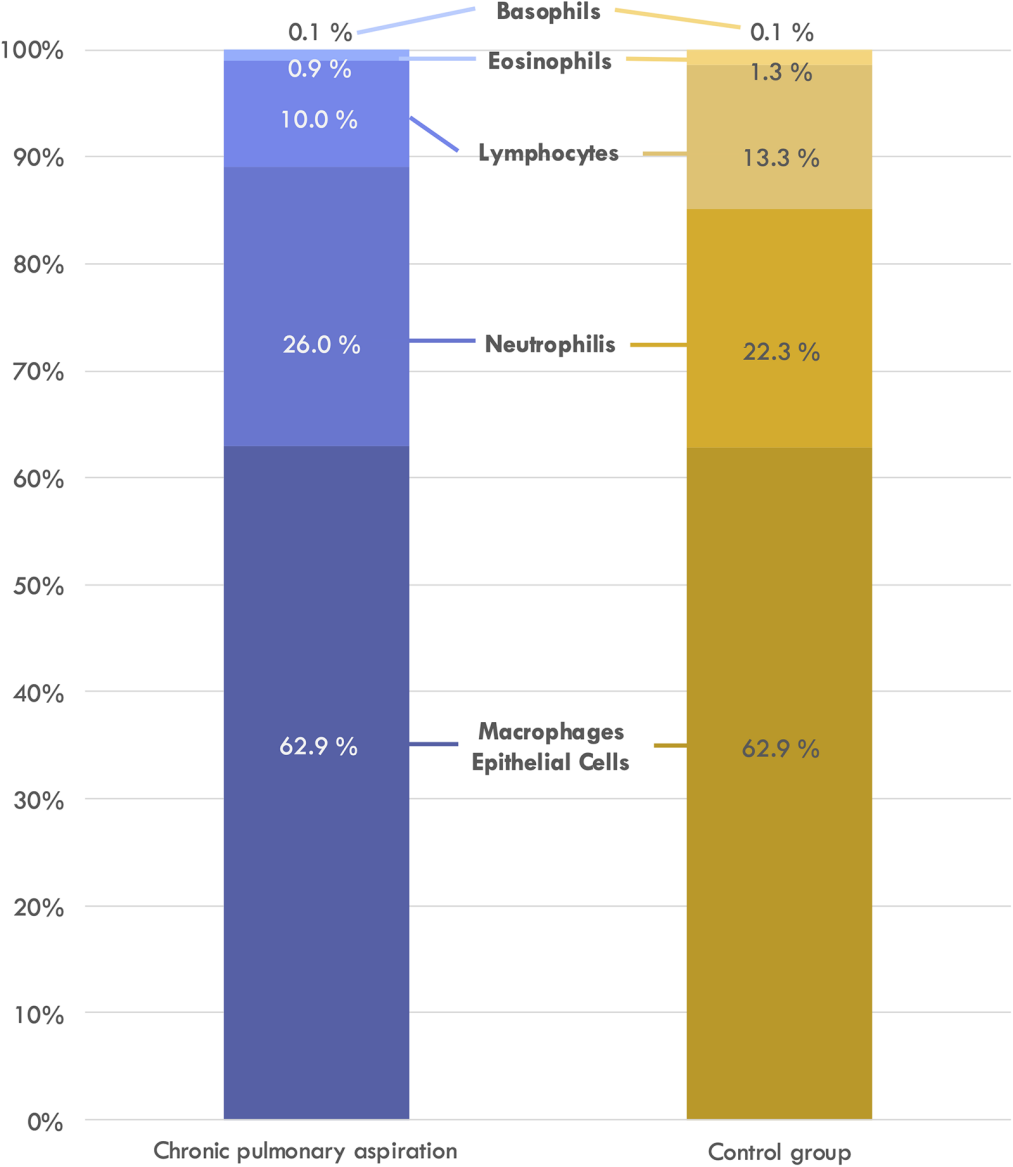
Relative proportion of white blood cell subgroup in the bronchoalveolar lavage.

The comparison between relative white blood cell proportions is displayed in Figure 3.

A statistical comparison between both groups was performed using student's *t*-test (comparison of means) and Chi-Square Test of independence (comparing number of cases with altered cell counts). As shown in Table 3, while total white blood cell counts were significantly elevated in the CPA group compared to controls (*p* = 0.022), analysis of leukocyte subpopulations revealed no significant differences in the relative proportions of neutrophils, lymphocytes, macrophages, eosinophils, or basophils between groups.

**Table 3:** Comparison of means and relative proportions that deviate from the reference range between white blood cell (WBC) populations in the BALF of patients with and without swallowing disorder.

Cell population		CPA group			Control group			Standard	*T*-test	*χ*^2^ test		
Type of cells	Unit of measurement	*N*	Mean	Proportion of cases outside the reference range (%)	*N*	Mean	Proportion of cases outside the reference range (%)	Reference range	2-sided *P*-value	χ^2^ value	df	2-sided *P*-value
WBC	/μl	45	813.7	88.9	297	623.8	26.9	<100	.494	5.239	1	.022*
Macrophages/epithelial cells	%	48	62.9	50	320	62.51	50.3	>75	.927	0.002	1	.968
Lymphocytes	%	48	10.0	18.8	319	13.3	17.2	<21	.088	0.066	1	.838
Neutrophilic granulocytes	%	48	26.0	77.1	318	22.27	70.4	<3	.419	0.900	1	.395
Basophilic granulocytes	%	43	0.05	4.7	271	.13	10.0	<1	.055	1.249	1	.396
Eosinophilic granulocytes	%	47	0.94	29.8	299	1.33	38.8	<1	.442	1.405	1	.260

WBC, White Blood Cells; df, degrees of freedom. Student’s *T*-Test for independent samples was performed to compare the mean number of WBC populations, χ^2^ test for independence was performed to compare ratio of cases with elevated or decreased cell populations.

* Significant *P*-values

## 4 Discussion

his study demonstrates that children with chronic pulmonary aspiration (CPA) exhibit a distinct microbial composition in their lungs compared to those without swallowing difficulties. We found that *Enterobacterales* and *Pseudomonas aeruginosa* were significantly more prevalent in children with CPA, occurring in approximately 10% of these patients, compared to 2% and 1%, respectively, in those without swallowing difficulties. These findings highlight the role of pathogenic bacterial colonization in the pathogenesis of CPA, which may contribute to long-term lung damage.

Although culture-based data for children with swallowing disorders is limited, our results align with previous studies that have found varying microbial profiles in pediatric respiratory diseases. For example, Andereson reported *Enterobacterales* in 7% and *P. aeruginosa* in 15% of children with diverse lung conditions (10) while Baets et al. detected *Enterobacterales* in 5% and *P. aeruginosa* in 1% of infants with persistent respiratory symptoms (11). However, comparing these findings across studies is challenging due to variations in sampling techniques, underlying conditions, and the inherent invasiveness of obtaining bronchoalveolar lavage fluid (BALF) from healthy children.

Beyond these characteristic pathogens, our investigation also revealed important patterns regarding antimicrobial resistance. Although multi-drug resistant (MDR) organisms were detected more frequently in CPA patients (20.8%) compared to controls (3.1%), this difference was not statistically significant. Nevertheless, the presence of 3MRGN and 4MRGN bacteria in children with CPA merits clinical attention. These patients often receive multiple antibiotic courses for recurrent respiratory infections, potentially increasing their risk for resistant organism colonization. The detection of one 4MRGN organism in the CPA group underscores the importance of antimicrobial stewardship in this vulnerable population. Further research with larger cohorts is needed to establish whether specific surveillance or modified empiric treatment protocols would benefit these patients, especially as antimicrobial resistance remains a growing global concern.

An intriguing finding in our study was the elevation of total white blood cell counts in BALF from CPA patients without a corresponding increase in neutrophil proportions. This contrasts with previous smaller studies that reported neutrophilia as a characteristic feature of aspiration-related inflammation (6, 8). This discrepancy may be explained by several factors: the heterogeneity of aspirated material (food, saliva, or gastric contents) triggering variable immune responses; the intermittent rather than continuous nature of aspiration events; and our cohort's diverse composition, including patients at various stages of lung disease. Additionally, underlying conditions and medications in our CPA cohort may modify immune responses. This finding emphasizes the complex inflammatory pattern in CPA and suggests that neutrophil-dominated inflammation may not be universal across all aspiration phenotypes or disease stages.

Our study applied a standardized BALF sampling method across all patients to minimize potential bias, ensuring comparability within the cohort. Interestingly, bacteria from the oral and pharyngeal microbiota were present in nearly 90% of patients, irrespective of swallowing function. This contrasts with previous findings by Vielkind et al., who observed a higher prevalence of mixed upper respiratory flora and pathogen colonization in children with dysphagia, particularly those with cerebral palsy (6). Additionally, while their study found elevated neutrophil counts in dysphagic patients, our data suggest that the presence of potential pathogens, rather than dysphagia alone, correlates with increased neutrophils and reduced macrophages across all patients.

This study has several limitations. First, as a retrospective observational study, it lacks the control and robustness of prospective trials, limiting its ability to establish causality. Second, the heterogeneity of our control group, which included children with a wide range of underlying conditions, complicates direct comparisons. Third, culture-based methods may miss important bacterial species, and more advanced techniques like 16S rRNA sequencing or next-generation sequencing would provide a more comprehensive analysis of microbial diversity (12).

In conclusion, our findings provide new insights into the microbiome of children with CPA, suggesting that altered microbial colonization may contribute to disease progression. These results point toward potential therapeutic strategies, including targeted antibiotic approaches for *Enterobacterales* and *P. aeruginosa* eradication, microbiota-modulating interventions to restore beneficial communities, and prophylactic measures for high-risk patients. Future large-scale, prospective studies should address these limitations through more homogeneous control groups, metagenomic sequencing for comprehensive microbiome characterization, longitudinal sampling to establish causality, and correlation of microbiological findings with clinical outcomes to optimize treatment protocols for this vulnerable population.

## Data Availability

The original contributions presented in the study are included in the article/Supplementary Material, further inquiries can be directed to the corresponding author.
