# Supplementary material for: Microbial alterations in the lungs of children with chronic pulmonary aspiration
Source: Front Pediatr. 2025 Apr 11;13:1520487. doi: 10.3389/fped.2025.1520487 (PMC12021908; doi:10.3389/fped.2025.1520487)
Supplement: Supplementary file 1 [file Supplementaryfile1.docx]

Supplementary Material

# Supplementary Figure

##
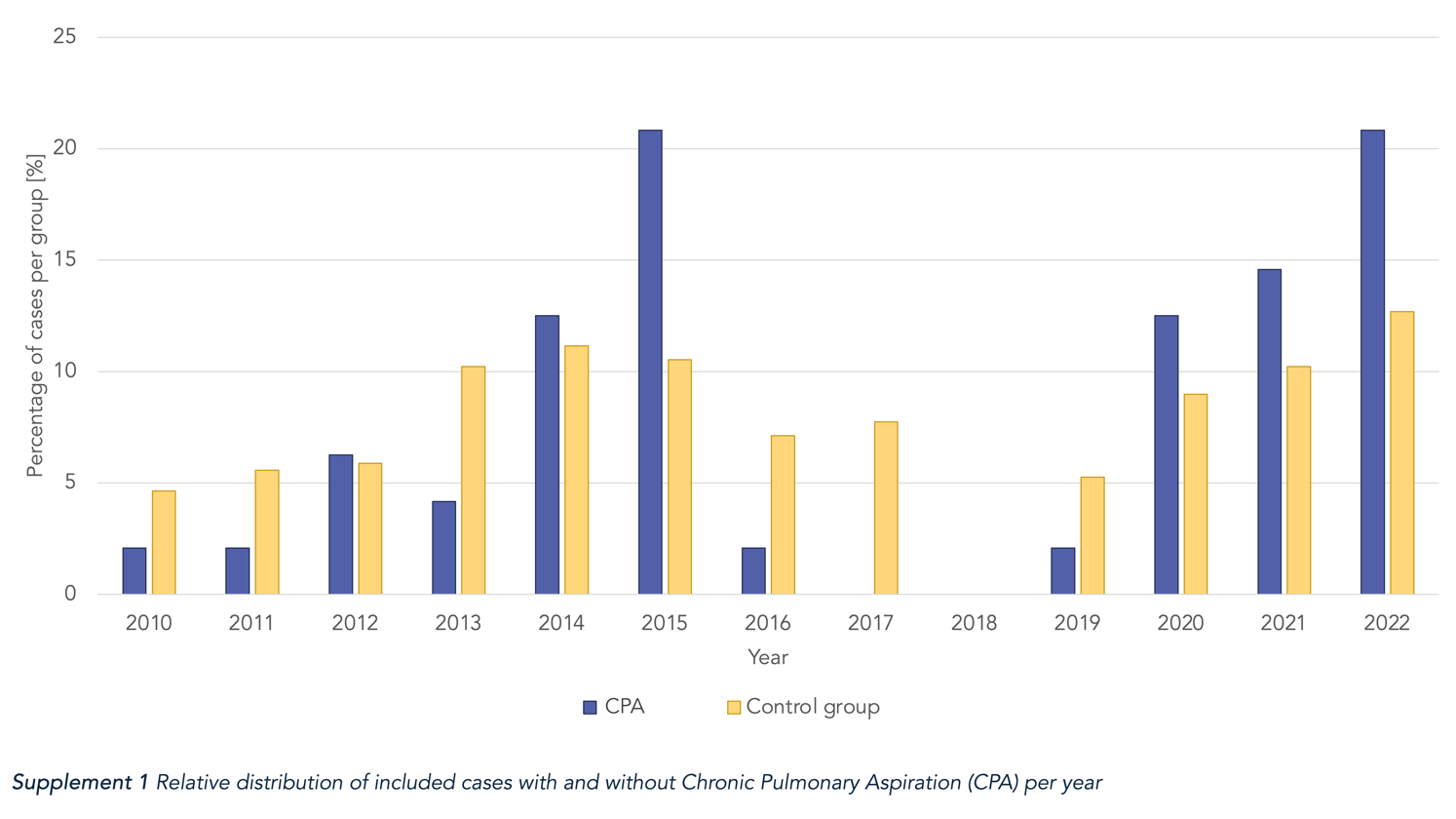


**Supplementary Figure 1** Relative distribution of included cases with and without Chronic Pulmonary Aspiration (CPA) per year.

# Supplementary Tables

## Supplementary Table 1

| **Supplement Table 1.** Bronchoscopic findings of airway anatomy | | | | | | | | | | | | |
| --- | --- | --- | --- | --- | --- | --- | --- | --- | --- | --- | --- | --- |
|  | | |  | Dysphagia group (n=48) | | |  | Control group (n=323) | | |  |  |
|  | | |  | n |  | % |  | n |  | % |  | p-value^+^ |
| **Altered airway anatomy** | | |  | 26 |  | 54.17 |  | 101 |  | 31.27 |  | **.002*** |
|  | Cleft | |  | 2 |  | 4.17 |  | 0 |  | 0.00 |  | - |
|  | Hemangioma | |  | 0 |  | 0.00 |  | 0 |  | 0.00 |  | - |
|  | Cyst | |  | 0 |  | 0.00 |  | 1^#^ |  | 0.31 |  | - |
|  | Papilloma | |  | 0 |  | 0.00 |  | 2 |  | 0.62 |  | - |
|  | Fistula | |  | 1^$^ |  | 2.08 |  | 1^$^ |  | 0.31 |  | .117 |
|  | Aberrant tracheal bronchus | |  | 1 |  | 2.08 |  | 6 |  | 1.86 |  | .915 |
|  | **Location:** | |  |  |  |  |  |  |  |  |  |  |
|  | | Trachea |  | 8 |  | 16.67 |  | 34 |  | 10.53 |  | .210 |
|  | | Right middle lobe |  | 3 |  | 6.25 |  | 29 |  | 8.98 |  | .530 |
|  | | Left main stem bronchus |  | 3 |  | 6.25 |  | 14 |  | 4.33 |  | .554 |
|  | | Larynx |  | 14 |  | 29.17 |  | 12 |  | 3.72 |  | **< .001*** |
|  | | Right upper lobe |  | 2 |  | 4.17 |  | 9 |  | 2.79 |  | .600 |
|  | | Left lower lobe |  | 1 |  | 2.08 |  | 7 |  | 2.17 |  | .970 |
|  | | Right main stem bronchus |  | 4 |  | 8.33 |  | 6 |  | 1.86 |  | **.009*** |
|  | | Segment 7 |  | 0 |  | 0.00 |  | 3 |  | 0.93 |  | - |
|  | | Right lower lobe |  | 0 |  | 0.00 |  | 3 |  | 0.93 |  | - |
|  | | Generalised |  | 0 |  | 0.00 |  | 3 |  | 0.93 |  | - |
|  | | Segment 1 |  | 0 |  | 0.00 |  | 2 |  | 0.62 |  | - |
|  | | Segment 4 |  | 0 |  | 0.00 |  | 2 |  | 0.62 |  | - |
|  | | Segment 6 |  | 1 |  | 2.08 |  | 2 |  | 0.62 |  | .291 |
|  | | Left upper lobe |  | 4 |  | 8.33 |  | 2 |  | 0.62 |  | **< .001*** |
|  | | Segment 5 |  | 0 |  | 0.00 |  | 1 |  | 0.31 |  | - |
|  | | Right lung |  | 0 |  | 0.00 |  | 1 |  | 0.31 |  | - |
| ^#^Right stem bronchus. ^$^Trachea. ^+^p-value was computed by Chi-Square test for independence | | | | | | | | | | | | |

## Supplementary Table 2

| **Supplement Table 2.** Bronchoscopic signs of inflammation | | | | | | | | | | | | |
| --- | --- | --- | --- | --- | --- | --- | --- | --- | --- | --- | --- | --- |
|  | | |  | Dysphagia group (n=48) | | |  | Control group (n=323) | | |  |  |
|  | | |  | n |  | % |  | n |  | % |  | p-value^+^ |
| **Inflammation present** | | |  | 28 |  | 58.33 |  | 213 |  | 65.94 |  | .302 |
|  | Redness | |  | 7 |  | 14.58 |  | 124 |  | 38.39 |  | **.001*** |
|  | Hypersecretion | |  | 13 |  | 27.08 |  | 114 |  | 35.29 |  | .263 |
|  | “Pathway formation” | |  | 2 |  | 4.17 |  | 52 |  | 16.10 |  | **.029*** |
|  | **Location:** | |  |  |  |  |  |  |  |  |  |  |
|  | | Generalised |  | 23 |  | 47.92 |  | 153 |  | 47.37 |  | .636 |
|  | | Left main stem bronchus |  | 2 |  | 4.17 |  | 29 |  | 8.98 |  | .261 |
|  | | Trachea |  | 1 |  | 2.08 |  | 21 |  | 6.50 |  | .227 |
|  | | Right main stem bronchus |  | 1 |  | 2.08 |  | 19 |  | 5.88 |  | .277 |
|  | | Right lung |  | 4 |  | 8.33 |  | 14 |  | 4.33 |  | .229 |
|  | | Left lung |  | 4 |  | 8.33 |  | 12 |  | 3.72 |  | .142 |
|  | | Right middle lobe |  | 1 |  | 2.08 |  | 3 |  | 0.93 |  | .470 |
|  | | Larynx |  | 1 |  | 2.08 |  | 3 |  | 0.93 |  | .470 |
|  | | Recht upper lobe |  | 0 |  | 0.00 |  | 1 |  | 0.31 |  | - |
|  | | Right lower lobe |  | 0 |  | 0.00 |  | 1 |  | 0.31 |  | - |
|  | | Left upper lobe |  | 0 |  | 0.00 |  | 1 |  | 0.31 |  | - |
|  | | Left lower lobe |  | 0 |  | 0.00 |  | 1 |  | 0.31 |  | - |
|  | | Segment 8 |  | 1 |  | 2.08 |  | 0 |  | 0.00 |  | - |
| ^+^p-value was computed by Chi-Square test for independence | | | | | | | | | | | | |

## Supplementary Table 3

| **Supplement Table 3.** Bronchoscopic findings of airway stability | | | | | | | | | | | |
| --- | --- | --- | --- | --- | --- | --- | --- | --- | --- | --- | --- |
|  | |  | Dysphagia group (n=48) | | |  | Control group (n=323) | | |  |  |
|  | |  | n |  | % |  | n |  | % |  | p-value^+^ |
| **Airway instability** | |  | 24 |  | 50.00 |  | 115 |  | 36.6 |  | .055 |
|  | Larnygomalacia |  | 8 |  | 50.00 |  | 27 |  | 8.36 |  | .066 |
|  | Subglottic stenosis |  | 3 |  | 16.67 |  | 6 |  | 1.86 |  | .065 |
|  | Tracheomalacia |  | 6 |  | 6.25 |  | 14 |  | 4.33 |  | **.019** |
|  | Bronchomalacia (both sides) |  | 8 |  | 12.50 |  | 56 |  | 17.34 |  | .909 |
|  | Bronchomalacia (only right bronchus) |  | 2 |  | 16.67 |  | 12 |  | 3.72 |  | .878 |
|  | Bronchomalacia (only left bronchus) |  | 3 |  | 4.17 |  | 24 |  | 7.43 |  | .769 |
|  | Endoscopic signs of reflux |  | 12 |  | 6.25 |  | 41 |  | 12.69 |  | .023 |
| ^+^p-value was computed by Chi-Square test for independence | | | | | | | | | | | |
